# Spatial Distribution of *Bactrocera dorsalis* and *Thaumatotibia leucotreta* in Smallholder Avocado Orchards along Altitudinal Gradient of Taita Hills and Mount Kilimanjaro

**DOI:** 10.3390/insects9020071

**Published:** 2018-06-19

**Authors:** James J. Odanga, Samira Mohamed, Sizah Mwalusepo, Florence Olubayo, Richard Nyankanga, Fathiya Khamis, Ivan Rwomushana, Tino Johansson, Sunday Ekesi

**Affiliations:** 1 ICIPE-International Centre of Insect Physiology and Ecology, P.O. Box 30772-00100, Nairobi, Kenya; sfaris@icipe.org (S.M.); mwalusepo@yahoo.com (S.M.); fkhamis@icipe.org (F.K.); tino.johansson@helsinki.fi (T.J.); sekesi@icipe.org (S.E.); 2 Department of Plant Science and Crop Protection, University of Nairobi, Kenya, P.O. Box 30197-00100, Nairobi, Kenya; fmmogi@yahoo.com (F.O.); richardnyankanga@yahoo.com (R.N.); 3 Invertebrate Zoology Section, National Museums of Kenya, P.O. Box 40658-00100, Nairobi, Kenya; 4 Department of General Studies, Dar es Salaam Institute of Technology, P.O. Box 2958, Dar es Salaam, Tanzania; 5 CABI-Centre for Agriculture and Biosciences International, Africa Regional Centre, P.O. Box 633-000621, Nairobi, Kenya; i.rwomushana@cabi.org; 6 Department of Geosciences and Geography, University of Helsinki, P.O. Box 68, FI-00014 Helsinki, Finland

**Keywords:** avocado fruits, *Bactrocera dorsalis*, East African highlands, elevation, distribution, *Thaumatotibia leucotreta*

## Abstract

Avocado (*Persea americana*) fruits are an important source of income and a nutritious food for small-scale growers and other stakeholders involved in farming along the Afrotropical highlands of Taita Hills and Mount Kilimanjaro in Kenya and Tanzania, respectively. Avocado fruits are infested by several insect pests, namely the Asian invasive fruit fly, *Bactrocera dorsalis* (Hendel) (Diptera: Tephritidae), and the false codling moth, *Thaumatotibia leucotreta* Meyrick (Lepidoptera: Tortricidae). However, there is inadequate information on the distribution patterns of these pests in small-scale avocado cropping systems in the East African highlands. This study was initiated to generate a spatial distribution map of *B. dorsalis* and *T. leucotreta* in avocado orchards at Taita Hills and Mount Kilimanjaro in Kenya and Tanzania, respectively. The two pests were monitored by using their respective parapheromone lures for two years between August 2012 and July 2014. Fruit damage was assessed by computing the proportion of infested fruits for *B. dorsalis*, whereas the damage score was used for *T. leucotreta*. Our results indicated that the mean number of *B. dorsalis* per trap per day differed significantly across elevation, being highest in lowland zone for both Taita Hills (15.90) and Mount Kilimanjaro (24.45). Similarly, the percentage infestation of ground collected fruits by *B. dorsalis* varied with altitude, being lowest at highlands above 1500 m.a.s.l. (0.66% and 0.83% for Taita Hills and Mount Kilimanjaro, respectively). Conversely, the mean number of *T. leucotreta* did not vary with altitude in either study area. However, the damage score for *T. leucotreta* infestation was significantly lower in the highlands of both transects (7.0% and11.1% for Taita Hills and Mount Kilimanjaro, respectively). These findings describe spatial trends that are important in formulating strategies aimed at suppressing the populations of *B. dorsalis* and *T. leucotreta* in East African avocado cropping systems.

## 1. Introduction

Kenya is one of the major avocado-growing countries in Africa, with an annual production of 186,292 metric tonnes, while Tanzania witnessed a 20% growth in avocado production between 2005 and 2012 [1,2,3,4,5,6]. In both countries, avocado fruits are an important source of income and a nutritious food [7,8,9] for the smallholder growers and other stakeholders involved in farming and production [3,6]. However, avocado fruits are infested by several insect pests, specifically the false codling moth, *Thaumatotibia leucotreta* Meyrick (Lepidoptera: Tortricidae), and Asian invasive fruit fly, *Bactrocera dorsalis* (Hendel) (Diptera: Tephritidae), leading to phytosanitary restrictions by importing countries [10,11,12,13,14,15].

*Bactrocera dorsalis* and *Thaumatotibia leucotreta* cause tremendous yield losses of various fruit crops including avocadoes [10,11,12,16,17,18,19]. Both *B. dorsalis* and *T. leucotreta* are considered A1 quarantine pests because of the enormous losses they inflict due to direct infestation of fruits and quarantine restrictions imposed by importing countries [19,20,21]. For example, the invasion of *B. dorsalis* in Kenya and Tanzania led to a loss of the avocado export market to South Africa in 2007, causing revenue losses of US$1.9 million [20] with cumulative losses amounting to $15.2 million by the end of 2014 [12]. Considering the socioeconomic and nutritional significance of the avocado crop and the magnitude of losses caused by *B*. *dorsalis* and *T. leucotreta*, there is an urgent need for suppression of these pests to enhance quality fruit yield. Such efforts will not only improve the livelihood of the smallholder growers, who account for more than 70% of avocado producers in East Africa [3], but also help the governments of Kenya and Tanzania to regain full access to the lucrative export markets. A prerequisite for such intervention is a sound knowledge of the abundance and distribution patterns of these pests. However, only scanty information is available for commercial or large-scale avocado farming systems in East Africa [11,21]. This study was initiated to establish the spatial distributions of *B. dorsalis* and *T. leucotreta* in small-scale avocado cropping systems at Taita Hills in southeastern Kenya and Mount Kilimanjaro in northeastern Tanzania. This study had the following objectives: (i) Assess percentage damage levels in mature avocado fruits by *B. dorsalis* and *T. leucotreta* along elevational gradient of Taita Hills and Mount Kilimanjaro; (ii) Describe changes in the population density of *B. dorsalis* and *T. leucotreta* along an altitudinal gradient at the two transects and (iii) Determine how environmental variables influence changes in population density of the two pest species in each study area.

## 2. Materials and Methods

### 2.1. Study Areas and Agro-Ecological Conditions

**Figure 1:**
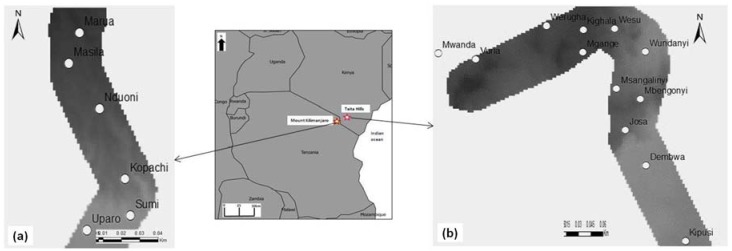
Location of study areas along the southeastern slopes of Mount Kilimanjaro in northeastern Tanzania and the Taita Hills in southeastern Kenya. White circles are sampling blocks along each study transect. (**a**) Mount Kilimanjaro; (**b**) Taita Hills.

This study was carried out along an altitudinal gradient between 900 and 1800 m.a.s.l. in small-scale avocado orchards at Taita Hills in southeastern Kenya and Mount Kilimanjaro in northeastern Tanzania (Figure 1). The two study transects were selected based on the criteria that they represent a steep elevation gradient with no large commercial farming that would influence the land use patterns and interactions between the lowland and highland environments [22,23]. Furthermore, farming along the two study transects is rain-fed and the smallholder avocado growers do not use any form of pesticide to control insect pests. The Taita Hills are located between 03°481′ S, 38°378′ E and 03°402′ S, 38°296′ E. The average monthly rainfall of the Taita Hills transect is 135.19 mm, with a mean humidity of 81.46% and mean temperature of 19.56 °C. Avocado plants are the dominant crop within the farmlands at Taita Hills that are intertwined between relics of the original cloud forests, forming a strong agro-ecosystem along the study area [24]. The Mount Kilimanjaro transect is located between 03°378′ S, 37°450′ E and 03°481′ S, 37°456′ E. The average monthly rainfall at Mount Kilimanjaro is 107.83 mm, with a mean relative humidity and temperature of 78.97% and 20.14 °C, respectively. The study area along the slopes of Mount Kilimanjaro is homogenous farmland, with avocado trees being the dominant fruit crop.

Three distinct agro-ecological zones were selected at each transect: lowland sector (900–1199 m.a.s.l.), mid-transition (1200–1499 m.a.s.l.) and highlands (1500–1799 m.a.s.l.), all dominated by avocado crop farming (Table 1). Each agro-ecological zone in a study transect was divided into five study blocks. A study transect comprised a total of 15 blocks, with each block consisting of at least 100 avocado trees. Temperature (°C) and relative humidity (%) were recorded daily at each block using iButton™ data loggers (Maxim Integrated Products, San Jose, CA, USA). The data loggers were hung in the lower canopy of avocado trees at a height of 2 m. Rainfall records was obtained from the weather stations set along the study transects in Kenya and Tanzania. The geographical coordinates and elevation of every study site in a block were recorded using a hand-held Garmin GPS eTrex 30 receiver (Garmin International, Olathe, KS, USA).

### 2.2. Insect Monitoring Using Lures

*Bactrocera dorsalis* and *Thaumatotibia leucotreta* were monitored along an elevational gradient between August 2012 and July 2014 in each of 15 blocks at each transect, namely Taita Hills and Mount Kilimanjaro. In every block three avocado trees were randomly selected for each target insect pest. For *B*. *dorsalis*, one Lynnfield trap baited with methyl eugenol (ME) charged on a cotton wick at a ratio of 4:1 of the ME: othothion as a killing agent was hung on each of the selected trees approximately 2 m above the ground. On the other three trees, yellow delta traps baited with dodecenyl acetate lure were used for monitoring *T. leucotreta*. The traps were hung at the same height as described for *B*. *dorsalis*. During the two years, traps for *B. dorsalis* and *T. leucotreta* were randomly rotated every week among 100 mature avocado trees in each block, but they were never concurrently hung on the same tree to avoid cross-contamination.

### 2.3. Fruits Damage Assessment

Five avocado trees were randomly selected from each of the 15 blocks for damage assessment of their fruits. Four fruits, two from the ground and two from the tree, were collected during the peak avocado harvesting season (May to June) and processed according to a protocol described by Ekesi et al. [25]. The collected fruits were transported to a temporary laboratory established in lowland area at each transect. The fruits were kept individually in plastic containers with a layer of heat-sterilized sand. Thereafter, the pupae yield from fruit of each block were collected in a Petri dish and placed in a small cage (30 × 30 × 30 cm) until the emergence of flies. The percentage of fruits infested by *B*. *dorsalis* was computed for each block and, thereafter, the emerged fruit flies were killed to reduce possible re-infestation. The percentage of infested fruits by *T. leucotreta* was based on the damage symptoms rather than the actual larval count, since the larvae leave the avocado fruits before maturation [26]. Therefore, infestation by *T. leucotreta* was recorded on mature fruits collected from the tree during the harvesting season. Twenty physiologically mature fruits were collected from each block along both transects. In the laboratory, fruits were examined for the typical triangle black scars on the exocarp of avocado fruit caused by the larvae of false codling moth, as described by Du Toit et al. [26].

### 2.4. Statistical Analysis

Data on insect trapping and fruit damage for both of *B. dorsalis* and *T. leucotreta* were analysed using Wilcoxon and Kruskal–Wallis Chi-square tests. A Wilcoxon signed rank test was employed to analyse differences between pairs of datasets, whereas a Kruskal–Wallis Chi-square test was used to evaluate differences among three groups of datasets, as described by Crawley and Versani [27,28,29]. Linear mixed effect (LME) models were used to determine the environmental variables that best explained the changes in population density of *B. dorsalis* and *T. leucotreta* along an altitudinal gradient in the two study areas [30]. All possible models were constructed based on sets of sampled explanatory (environmental) variables, and model evaluation was done using Akaike Information Criterion (AIC). The environmental variables used in analysis were mean temperature, average relative humidity, mean annual rainfall, elevation and agro-ecological zones of the two study areas. AIC is based on information theory and evaluates models according to model fit and complexity [28,29,30,31,32,33,34,35]. The best model for each transect was selected, and its statistical significance was determined based on classical hypothesis testing [34].

## 3. Results

**Figure 2:**
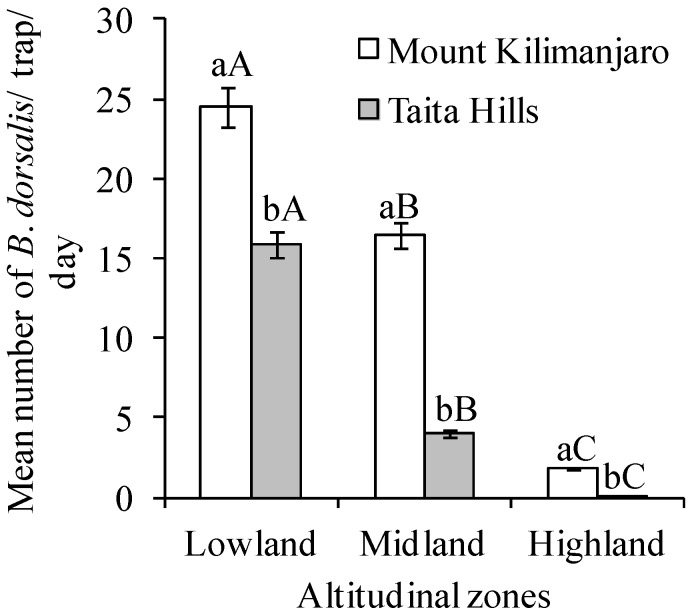
Number of *Bactrocera dorsalis* (Mean ± SE) along altitudinal zones of Taita Hills and Mount Kilimanjaro transects. Bars capped with the different lowercase letters within the same altitudinal zone are significantly different, while bars capped with different uppercase letters for the each transect are significantly different. The zones were categorized as lowland (900–1199 m.a.s.l.), midland (1200–1499 m.a.s.l.) and highlands (1500–1799 m.a.s.l.) regions.

**Figure 3:**
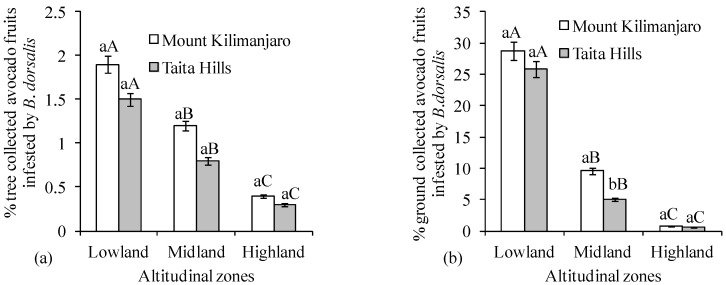
Percent infestation (Mean ± SE) by *Bactrocera dorsalis* of avocado fruits (**a**) picked from the tree and (**b**) collected from the ground along altitudinal zones of Taita Hills and Mount Kilimanjaro transects. Bars capped with the same lowercase letters within the same altitudinal zone are not significantly different, while bars capped with similar uppercase letters for the same transect are not significantly different.

The mean number of *B*. *dorsalis* per trap per day was different across elevation for both transects, Taita Hills (χ^2^ = 137.03, df = 2, *p* < 0.0001) and Mount Kilimanjaro (χ^2^ = 7.12, df = 2, *p* = 0.03), being highest at the lowland zone (15.90 ± 1.5 and 24.45 ± 2.9 *B*. *dorsalis*/trap/day for Taita Hills and Mount Kilimanjaro, respectively) (Figure 2). Comparing the same altitudinal zone of the two transects, *B*. *dorsalis* was more abundant at Mount Kilimanjaro for all altitudinal zones (V = 1416, *n* = 60, *p* = 0.0002; V = 1647, *n* = 60, *p* < 0.0001 and V = 1540, *n* = 60, *p* < 0.0001 for low, medium, and high elevations, respectively) (Figure 2). This was further substantiated by the results of fruit infestation percentage, which also varied dramatically with elevation for both transects, whether the fruits were picked from trees (χ^2^ = 66.09, df = 2, *p* < 0.0001 and χ^2^ = 44.78, df = 2, *p* < 0.0001 for Taita Hills and Mount Kilimanjaro, respectively) or collected from the ground (χ^2^ = 58.50, df = 2, *p* < 0.0001 and χ^2^ = 102.19, df = 2, *p* < 0.0001 for Taita Hills and Mount Kilimanjaro, respectively) (Figure 3a,b). Comparing fruits damaged by *B*. *dorsalis* in the same altitudinal zone of the two transects, the percent infestation was comparable for all agro-ecological zones for both fruits picked from the tree (V = 1069.5, *n* = 60, *p* = 0.2537; V = 357, *n* = 60, *p* = 0.1581 and V = 353, *n* = 60, *p* = 0.1469 for low, medium, and high elevations, respectively; Figure 3a) and ground collected (V = 1098, *n* = 60, *p* = 0.1771 and V = 294, *n* = 60, *p* = 0.8056 for low and high elevations, respectively; Figure 3b), except for the fruits collected from the ground at the middle elevation, whereby it was significantly higher at Mount Kilimanjaro transect (V = 1071, *n* = 60, *p* = 0.0045; Figure 3b).

**Figure 4:**
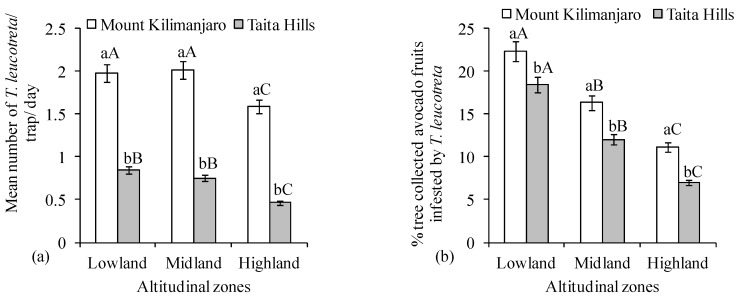
(**a**) Number of *Thaumatotibia leucotreta* (Mean ± SE) along agro-ecological zones of Taita Hills and Mount Kilimanjaro transects and (**b**) percent infestation of avocado fruits picked from the tree by *Thaumatotibia leucotreta*. Bars capped with the same lowercase letters within the same altitudinal zone are not significantly different, while bars capped with similar uppercase letters for the same transect are not significantly different.

The population density of *T*. *leucotreta* as measured by the mean numbers the moth per trap per day was comparable across elevations for both Taita Hills (χ^2^ = 7.03, df = 2; *p* = 0.03) and Mount Kilimanjaro (χ^2^ = 7.02, df = 2, *p* = 0.02) (Figure 4a). Comparing the same altitudinal zone between the two transects, *T*. *leucotreta* population was much higher at Mount Kilimanjaro for all agro-ecological zones (V = 1830, *n* = 60, *p* < 0.0001; V = 1824, *n* = 60, *p* < 0.0001 and V = 1776, *n* = 60, *p* < 0.0001 for low, medium, and high elevations, respectively (Figure 4a). The damage score for *T*. *leucotreta* infestation varied with elevation for both transects (χ^2^ = 58.50, df = 2, *p* < 0.0001 and χ^2^ = 102.19, df = 2, *p* < 0.0001 for Taita Hills and Mount Kilimanjaro, respectively) (Figure 4b). Comparing the damage score caused by *T*. *leucotreta* between same agro-ecological zones of the two transects, the *T*. *leucotreta* damage was consistently higher at Mount Kilimanjaro for all ecological zones (V = 843, *n* = 60, *p* = 0.011; V = 1278, *n* = 60, *p* = 0.006 and V = 535.5, *n* =60, *p* = 0.042 for low, medium and high elevations, respectively) (Figure 4b).

The variation in *B*. *dorsalis* abundance along altitudinal gradient was best explained by temperature alone for both Taita Hills (AIC = 1176.41, *R^2^* = 0.5876, F_5,174_ = 52.07, *p* < 0.0001) and Mount Kilimanjaro (AIC = 1455.23, *R^2^* = 0.53, F_5,174_ = 41.97, *p* < 0.0001) (Table 2). Variation in population density of *T. leucotreta* along the two transects was best explained by a set of environmental variables including humidity, rainfall, temperature (AIC = −3.98, *R^2^* = 0.21, F_7,172_ = 7.85, *p* < 0.0001 and AIC = 453.37, *R^2^* = 0.41, F_6,173_ = 21.62, *p* < 0.0001 for Taita Hills and Mount Kilimanjaro, respectively) (Table 3).

## 4. Discussion

Trapping of fruit flies using methyl eugenol indicated that *B*. *dorsalis* is more abundant in lower elevations of both transects; this is understandable considering that *B*. *dorsalis* is a lowland resident pest [25,36,37,38]. For example, in Hawaii, along the slope of Maui volcano, Wong et al. [36] found that trap capture of this pest was negatively correlated with elevation. In more recent studies, Geurts et al. [39,40] reported that *B*. *dorsalis* is the dominant species at elevation below 1000 m.a.s.l. in the Morogoro region of Tanzania. Moreover, *B*. *dorsalis* was more abundant in all agro-ecological zones of Mount Kilimanjaro than their corresponding ones in the Taita Hills. A possible reason for this discrepancy between the two transects in term of *B*. *dorsalis* population could be the fact that the former transect is relatively warmer by at least 0.5 °C; though this may seem negligible, *B*. *dorsalis* being poikilothermic, this difference in temperature will have a significant influence on the pest population growth. Another explanation for the higher *B*. *dorsalis* population in Mount Kilimanjaro could be the availability of more mango trees, the primary host plant of *B*. *dorsalis* [10,41,42], in this transect, especially at the lower elevation regions [39,40]. The intertwined nature of avocado with indigenous cloud forests along the Taita Hills transect might have contributed to the reduced population of *B*. *dorsalis* in this transect, as the natural, undisturbed vegetation provide refuge for natural enemies of this pest, especially predators such as the African weaver ant (*Oecophylla longinoda*). Way and Khoo, Van Mele and Cuc, Van Mele and Chien and Duyck et al. [43,44,45,46,47,48] reported that indigenous habitats enhance numbers of natural enemies that enable natural control of pest populations. However, it is worth mentioning that parasitoids are very unlikely to have been a contributing factor in this scenario [49,50,51,52], as this alien pest did not have resident parasitoid species.

The variation of the *B*. *dorsalis* population, as shown in the traps’ catches, was reflected in fruit infestation levels: highest at lower elevations and quite low at higher elevations of both transects. Our findings concurred with those of Geurts et al. [39,40], who reported that the percentage of sub-tropical fruits infested with fruit flies was significantly lower at elevated zones of the Eastern Arc Mountains in central Tanzania. The avocado orchards at higher elevation (>1600 m.a.s.l.) of Mount Kilimanjaro and Taita Hills can be considered areas of low pest prevalence from which avocado can be exported to lucrative international markets. Additional research is, therefore, required to advance our knowledge of how global warming will influence the envisaged uphill expansion of *B*. *dorsalis* in East African avocado cropping systems.

The higher *B*. *dorsalis* trap catch at Mount Kilimanjaro did not necessary translate into more fruit infestation compared to that at a similar elevation in the Taita Hills, except for the ground-collected fruits at middle elevations. This finding suggested that *B*. *dorsalis* could be utilizing other neighbouring host fruits, such as mango and guava, which are more abundant at Mount Kilimanjaro. It was probably from these host crops that most of the flies were attracted to methyl-eugenol-baited traps, resulting in higher *B*. *dorsalis* catches in lowlands. However, the distribution of *B. dorsalis* along the altitudinal gradient was influenced most by temperature, although other weather variables, such as humidity and rainfall, also played a significant role. In both transects, infestation was higher on fallen avocado fruits than tree-collected ones, which concurs with the results of Ware et al. [21]. This implies that sanitation of avocado orchards can contribute significantly to suppression of *B. dorsalis*, an approach that is not practiced by small-scale growers, especially in the Taita Hills and Mount Kilimanjaro transects; instead, they consider fallen fruits as manure. Unlike smallholders, commercial avocado farmers embrace efficient pest control methods, including orchard sanitation. Orchard sanitation has been promoted as part of integrated pest management (IPM) for the suppression of *B*. *dorsalis* in East Africa by Ekesi et al. [12]. The same approach has been recommended in South Africa for the management of other species related to fruit flies, such as *Ceratitis cosyra* and *Ceratitis rosa* [19].

Unlike *B*. *dorsalis*, the population density of *T. leucotreta* was more or less similar across all agro-ecological zones for each transect, implying that the pest has a wider thermal tolerance. For example, Newton, Stotter and Terblanche, Stotter and Boardman et al. [53,54,55,56] reported that *T. leucotreta* thrives well in the warmer lowlands and colder uplands of South Africa, where it attacks both cultivated and wild plants. Although the abundance of *T. leucotreta* did not vary with altitude, Mount Kilimanjaro agro-ecological zones had higher trap catches and fruit infestation levels than their corresponding agro-ecological zones in the Taita Hills transect. This could be explained by the fact that in the former transect there is a high diversity and more availability of alternative hosts of *T. leucotreta*, such as maize, *Zea mays* (Poaceae), sorghum, *Sorghum bicolor* (Poaceae), citrus, *Citrus sinensis* (Rutaceae) and guava, *Psidium guajava* (Myrtaceae). Moreover, the nature of the avocado plantation in Taita Hills transect, being intertwined with the indigenous forest, may have provided refuge for natural enemies and competitors of *T. leucotreta*, resulting in a reduced population in this transect. For example, the egg parasitoid (*Trichogrammatoidea cryptophlebiae*) of *T. leucotreta* is reported to cause high levels of parasitism in citrus orchards in South Africa [19,57]. However, *Trichogrammatoidea cryptophlebiae* was not recovered from the samples of avocado fruits collected at the two transects during the current study; this needs to be investigated further in East Africa.

## 5. Conclusions

Our findings revealed that *B. dorsalis* preferred to infest ripened avocado fruits, which were ground-collected in warmer lowlands, suggesting that physiologically healthy unharvested fruits are not the preferred hosts in the Taita Hills or Mount Kilimanjaro. However, damage by *T. leucotreta* was observed on avocado fruits across all altitudinal zones. Hence, integrated control measures for *B. dorsalis* in avocado orchards should be concentrated in lowland and adjacent mid-altitudinal zones, whereas the management of *T. leucotreta* must be tackled in all altitudinal zones. The present findings can be used by policy makers in designing efficient integrated pest management strategies aimed at controlling *B. dorsalis* and *T. leucotreta* in order to enhance the livelihood of smallholder avocado farmers in East Africa. Finally, there is a need to establish local farmer-led stakeholder awareness committees to disseminate sustainable management strategies for controlling avocado insect pests in Kenya and Tanzania.

## Figures and Tables

**Table 1:** Annual weather (Mean) of agro-ecological zones along the study region. The data were averaged from information that was collected at the Taita Hills and Mount Kilimanjaro transects between August 2012 and July 2014.

Elevation Range (m.a.s.l.)	Agro-Ecological Zone	Mean Elevation (m.a.s.l.)	Mean Temperature (°C)	Relative Humidity (%)	Mean Rainfall (mm)	Peak Avocado Harvesting Months
1500–1799	Highland	1687.0	17.6	84.4	218.7	June & July
1200–1499	Midland	1381.4	19.9	81.1	93.6	May & June
900–1199	Lowland	1085.7	22.0	75.2	52.2	April & May

**Table 2:** Linear mixed effect models explaining change in abundance of *Bactrocera dorsalis* along agro-ecological zones of Taita Hills in Kenya and Mount Kilimanjaro in Tanzania.

**a: Models for Taita Hills**	**AIC**	***R^2^***	***p*-Value**	**Rank**
*Bactrocera dorsalis* mean density vs. interaction term (mean temperature by agro-ecological zones) was the best model	1176.4	0.588	<0.0001	1
*Bactrocera dorsalis* mean density vs. mean temperature, mean humidity, rainfall and agro-ecological zones	1185.3	0.578	<0.0001	2
*Bactrocera dorsalis* mean density vs. mean temperature, mean humidity, rainfall, elevation, distance and agro-ecological zones	1188.4	0.575	<0.0001	3
*Bactrocera dorsalis* mean density vs. mean temperature, mean humidity and agro-ecological zones	1190.8	0.563	<0.0001	4
*Bactrocera dorsalis* mean density vs. mean temperature and habitats without interaction term (mean temperature by agro-ecological zones)	1200.8	0.535	<0.0001	5
*Bactrocera dorsalis* mean density vs. mean humidity and agro-ecological zones	1223.0	0.475	<0.0001	6
**b: Models for Mount Kilimanjaro**	**AIC**	***R²***	***p* -Value**	**Rank**
*Bactrocera dorsalis* mean density vs. interaction term (mean temperature by agro-ecological zones) was the best model	1455.2	0.534	<0.0001	1
*Bactrocera dorsalis* mean density vs. mean temperature and habitats without interaction term (mean temperature by agro-ecological zones)	1485.8	0.441	<0.0001	2
*Bactrocera dorsalis* mean density vs. mean temperature, mean humidity and agro-ecological zones	1487.8	0.438	<0.0001	3
*Bactrocera dorsalis* mean density vs. mean temperature, mean humidity, rainfall and agro-ecological zones	1488.1	0.440	<0.0001	4
*Bactrocera dorsalis* mean density vs. mean temperature, mean humidity, rainfall, elevation, distance and agro-ecological zones	1490.0	0.439	<0.0001	5
*Bactrocera dorsalis* mean density vs. mean humidity and agro-ecological zones	1519.0	0.328	<0.0001	6

**Table 3:** Linear mixed effect models explaining change in abundance of *Thaumatotibia leucotreta* along agro-ecological zones at Taita Hills, Kenya and Mount Kilimanjaro, Tanzania.

**a: Models for Taita Hills**	**AIC**	***R^2^***	***p*-Value**	**Rank**
*Thaumatotibia leucotreta* mean density vs. mean temperature, mean humidity, rainfall, distance and elevation was the best model	−3.98	0.2112	<0.0001	1
*Thaumatotibia leucotreta* mean density vs. mean temperature, mean humidity, rainfall, distance and agro-ecological zones	−2.24	0.1993	<0.0001	2
*Thaumatotibia leucotreta* mean density vs. mean temperature, mean humidity, rainfall and agro-ecological zones	12.08	0.0899	0.0007	3
*Thaumatotibia leucotreta* mean density vs. mean temperature	15.65	0.0610	0.0004	4
*Thaumatotibia leucotreta* mean density vs. mean humidity and agro-ecological zones	24.92	0.0193	0.0925	5
*Thaumatotibia leucotreta* mean density vs. distance and agro-ecological zones	26.53	0.0234	0.0925	6
**b: Models for Mount Kilimanjaro**	**AIC**	***R^2^***	***p*-Value**	**Rank**
*Thaumatotibia leucotreta* mean density vs. mean temperature, mean humidity, rainfall and elevation was the best model	453.37	0.4087	<0.0001	1
*Thaumatotibia leucotreta* mean density vs. mean temperature and habitats without interaction term (mean temperature by agro-ecological zones)	461.77	0.3402	<0.0001	2
*Thaumatotibia leucotreta* mean density vs. mean temperature, mean humidity and agro-ecological zones	463.65	0.3389	<0.0001	3
*Thaumatotibia leucotreta* mean density vs. mean temperature, mean humidity, rainfall and agro-ecological zones	464.46	0.3353	<0.0001	4
*Thaumatotibia leucotreta* mean density vs. mean temperature excluding agro-ecological zones	468.90	0,2795	<0.0001	5
*Thaumatotibia leucotreta* mean density vs. mean humidity and agro-ecological zones	503.04	0.2098	<0.0001	6
*Thaumatotibia leucotreta* mean density vs. distance and agro-ecological zones	541.60	0.2097	0.0813	7
